# Comments: Influence of the prone position on a stretcher for pregnant women on maternal and fetal hemodynamic parameters and comfort in pregnancy

**DOI:** 10.6061/clinics/2019/e878

**Published:** 2019-04-02

**Authors:** Alexander E P Heazell, Peter Stone

**Affiliations:** I Maternal and Fetal Health Research Centre, School of Medical Sciences, Faculty of Biological, Medical and Human Sciences, University of Manchester, UKUK; II St. Mary's Hospital, Central Manchester University Hospitals NHS Foundation Trust, Manchester Academic Health Science Centre, Manchester, UK; III Department of Obstetrics and Gynecology, The University of Auckland, Auckland, New Zealand

We read the paper by Oliveira C et al. 1 describing the hemodynamic effects of different maternal positions on maternal and fetal cardiovascular and respiratory indices with interest. The authors observed no differences in the measured parameters (maternal heart rate, blood pressure, oxygen saturation, respiratory rate or fetal heart rate) between the left lateral position, Fowler's position and supine position, but they did observe reductions in the maternal respiratory rate and systolic blood pressure in Fowler's position compared with lying prone on the convex stretcher. The authors concluded that on this type of stretcher, the prone position was considered safe and comfortable for mothers and could improve oxygen saturation, reduce systolic blood pressure and reduce the respiratory rate.

The authors' findings regarding maternal position contradict much published studies, which demonstrate that compared to the left lateral position, a supine maternal position results in reduced maternal cardiac output 2, uterine blood flow 2,3, reduced fetal oxygenation as measured by reduced resistance in the middle cerebral artery 4, oxygen saturation as assessed in labor and an altered fetal behavioral state in late pregnancy 5. These differences in observations may be due to the relatively short exposure time of the fetus to alterations in maternal position, which was 6 minutes in the study by Oliveira et al. and 3-30 minutes in other studies. Importantly, if women are having treatment in a supine or prone position, they are likely to spend longer than 6 minutes in that position. Furthermore, several studies have also described a relationship between the maternal sleep position and late stillbirth, suggesting that being in one maternal position for a prolonged period can have adverse fetal consequences 6. Therefore, we encourage Oliveira et al. to study the effects of maternal position on maternal and fetal hemodynamics over a longer, more clinically relevant timescale before concluding that the prone position is safe or that other maternal positions do not have effects on maternal hemodynamic indices.
